# Effectiveness of a multi-strategy intervention in increasing the implementation of vegetable and fruit breaks by Australian primary schools: a non-randomized controlled trial

**DOI:** 10.1186/1471-2458-12-651

**Published:** 2012-08-13

**Authors:** Nicole Nathan, Luke Wolfenden, Andrew C Bell, Rebecca Wyse, Philip J Morgan, Michelle Butler, Rachel Sutherland, Andrew J Milat, Debra Hector, John Wiggers

**Affiliations:** 1 Hunter New England Population Health, Hunter New England Area Health Service, Newcastle, Locked Bag No. 10, Wallsend, NSW, 2287, Australia; 2 School of Medicine and Public Health, The University of Newcastle, Newcastle, NSW, 2308, Australia; 3 Priority Research Centre for Health Behavior, The University of Newcastle, Newcastle, NSW, 2308, Australia; 4 Hunter Medical Research Institute, Newcastle, NSW, 2300, Australia; 5 Priority Research Centre in Physical Activity & Nutrition, Faculty of Education & Arts, The University of Newcastle, Newcastle, NSW, 2308, Australia; 6 Centre for Epidemiology and Evidence, NSW Ministry of Health, North Sydney, NSW, 2060, Australia; 7 Physical Activity, Nutrition and Obesity Research Group, School of Public Health, The University of Sydney, Sydney, NSW, 2006, Australia

**Keywords:** Implementation, Primary schools, Fruit, Vegetables, Intervention, Dissemination, Diffusion

## Abstract

**Background:**

Limited evidence exists describing the effectiveness of strategies in facilitating the implementation of vegetable and fruit programs by schools on a population wide basis. The aim of this study was to examine the effectiveness of a multi-strategy intervention in increasing the population-wide implementation of vegetable and fruit breaks by primary schools and to determine if intervention effectiveness varied by school characteristics.

**Methods:**

A quasi-experimental study was conducted in primary schools in the state of New South Wales, Australia. All primary schools in one region of the state (n = 422) received a multi-strategy intervention. A random sample of schools (n = 406) in the remainder of the state served as comparison schools. The multi-strategy intervention to increase vegetable and fruit breaks involved the development and provision of: program consensus and leadership; staff training; program materials; incentives; follow-up support; and implementation feedback. Comparison schools had access to routine information-based Government support. Data to assess the prevalence of vegetable and fruit breaks were collected by telephone from Principals of the intervention and comparison schools at baseline (2006–2007) and 11 to 15 months following the commencement of the intervention (2009–2010). GEE analysis was used to examine the change in the prevalence of vegetable and fruit breaks in intervention schools compared to comparison schools.

**Results:**

At follow-up, prevalence of vegetable and fruit breaks increased significantly in both intervention (50.3 % to 82.0 %, p < 0.001) and comparison (45.4 % to 60.9 % p < 0.001) schools. The increase in prevalence in intervention schools was significantly larger than among comparison schools (OR 2.36; 95 % CI 1.60-3.49, p <0.001). The effect size was similar between schools regardless of the rurality or socioeconomic status of school location, school size or government or non-government school type.

**Conclusion:**

The findings suggest that a multi-strategy intervention can significantly increase the implementation of vegetable and fruit breaks by a large number of Australian primary schools.

## Background

Longitudinal studies have shown that intake of vegetables and fruit in childhood can reduce the subsequent development of chronic diseases, including cardiovascular disease and cancers [1-3]. Moreover, a diet high in vegetables and fruit provides immediate nutritional benefits for children as well as protection against obesity [4,5] and some respiratory diseases [6]. Despite this, children in many developed countries fail to consume adequate daily quantities of vegetables and fruit [7-9]. As a consequence, increasing children’s consumption of vegetables and fruit is a recognized public health priority [10], with the population wide implementation of effective programs promoting such behavior being a recommended chronic disease prevention strategy [10-12].

One recommended setting for promoting children’s vegetable and fruit consumption on a population-wide basis is schools [13]. Systematic reviews have consistently found that school-based vegetable and fruit programs are efficacious, and increase children’s daily vegetable and fruit consumption [13-20]. Consequently, a number of vegetable and fruit programs and schemes have been developed for implementation in schools. For example the United Kingdom [9], United States [21], Norway [22] and New Zealand [23] have school vegetable and fruit schemes which provide all or some students with a fully or partially subsidized piece of vegetable or fruit to consume each day at school. As an alternative to such distribution programs, Australian schools have been encouraged to implement a vegetable and fruit break program that provides a time in class for children to consume a piece of vegetable or fruit they have brought from home [24].

There have been calls for rigorous implementation research to identify the strategies that are effective in supporting population-wide implementation of such programs so that they benefit the health of the community [25]. The need for such research is illustrated by the findings of a recent update of an Agency for Health Care Policy and Research review published in 2010. The review of interventions in community based settings, including schools, primarily sought to evaluate the effectiveness of implementation or dissemination interventions on policies or programs promoting healthy eating, physical activity, sun protection, or preventing tobacco use. Implementation and dissemination studies were defined as those that evaluated the “effectiveness of efforts that enable the widespread use of an evidence-based intervention by the target population and its successful integration within a particular setting”. The review identified just four school based interventions to improve the adoption of healthy eating policies or practices in schools and none targeted the adoption of vegetable and fruit initiatives specifically [26].

However, theoretical frameworks regarding organizational change more broadly [27,28] and evidence from reviews and trials assessing the efficacy of implementation strategies addressing other health issues in schools [29-32], suggest that multi-strategy interventions that; develop the support of key opinion leaders, provide program materials and training, monitor program implementation, and provide technical support and implementation feedback, are most likely to be effective in changing service delivery practice. In the above mentioned review of implementation interventions in community settings [26], those that have adopted a multi-strategy approach have reported a change in prevalence of program implementation of between 50 %-78 %. However, only a few of these studies have targeted a large number (>100) of schools [33-36] or utilized evaluation designs incorporating comparison groups [34,35,37], limiting the ability to infer causality.

In order to achieve the potential public health benefits of school based vegetable and fruit programs, further evidence is required regarding the strategies that are effective in facilitating the implementation of such programs across an entire population of schools. In addition, as the implementation of programs by schools has been reported to be associated with school characteristics such as number of students, and socioeconomic and geographic characteristics, further evidence is required regarding the effectiveness of such strategies for different types of schools [38,39].

In this context we undertook a study to assess the effectiveness of a multi-strategy intervention, relative to information-based support, in increasing the implementation of an in-class vegetable and fruit break by a population of primary schools.

## Methods

### Design and setting

A quasi-experimental study was conducted in a large cohort of primary and central schools in the state of New South Wales, Australia. The multi-strategy intervention was delivered in the Hunter New England region of the state as part of a large child obesity prevention program (*Good for Kids. Good for Life.)*[40]. The Hunter New England region covers a large non-metropolitan area (more than 130 000 km^2^); with a demographically and socioeconomically diverse population of approximately 121 000 children aged 5–14 years (14 % of the state population of 5–14 year olds) [41]. Approval to conduct the study was obtained from the Hunter New England Area Health Service Human Research Ethics Committee (no. 06/07/26/4.04) and relevant school ethics committees.

### Sample and recruitment

A database of all primary schools (children 5–12 years of age) and central schools (children 5–18 years of age) across the state was generated from information provided by the New South Wales Department of Education and Communities [42] Catholic Education Office [43] and the Association of Independent Schools websites [44]. All schools were eligible to participate in the study other than special purpose schools catering for students with special needs, juvenile justice or schools serving children who are hospitalized.

**Figure 1:**
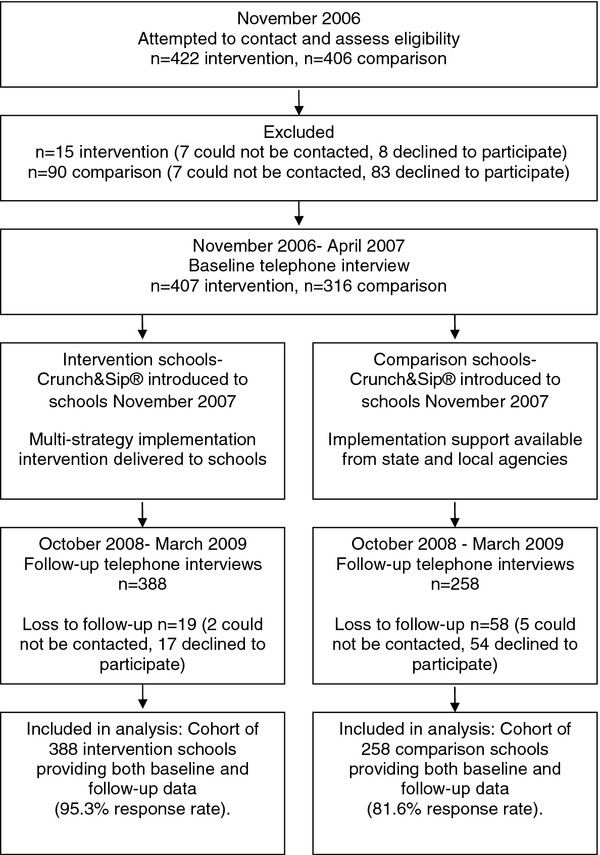
Study flow diagram.

All 422 of the eligible intervention schools were invited to receive the multi-strategy intervention and to participate in data collection (Figure 1). To serve as the comparison schools, 406 eligible schools from the rest of the state (23 %) were randomly selected to participate in data collection. Principals of both groups of schools were sent a letter inviting them to participate in the study. Two weeks after receipt of the letter, Principals were telephoned by a trained research assistant who confirmed school eligibility, sought consent to participate and scheduled a time for a telephone interview.

### Vegetable and fruit break program

A program designed to promote the consumption of vegetables and fruit during class time (*Crunch&Sip®*) [45], was made available to both intervention and comparison schools in November 2007. The program, which was promoted as part of the Australian Government’s “Go for 2&5®” campaign; a social marketing campaign to increase consumption of vegetables and fruit in the general population, required schools to implement time in class for children to eat a piece of vegetable or fruit that they had brought from home, and to drink water. In addition to implementing such breaks, the program required schools to: develop and endorse a school vegetable, fruit and water break policy; implement teaching and learning materials that reinforced the related key nutrition messages; and to advertise and promote the breaks to teachers, students and parents through newsletter articles, letters to parents and classroom posters.

### Implementation of vegetable and fruit breaks

#### Multi-strategy implementation intervention

To facilitate the implementation of vegetable and fruit breaks in the intervention schools, a structured multi-strategy intervention was developed based on theoretical frameworks of practice change [27] and recommendations from reviews and implementation studies conducted in schools and other settings [29-32]. Table 1 provides a detailed description of the intervention strategies. 

**Table 1:** Multi-strategy intervention to facilitate implementation of vegetable and fruit breaks in intervention schools

**Change strategy**	**Strategy components**
Consensus processes, leadership support & endorsement	· Memorandum of Understanding [27] signed with New South Wales Department of Education & Communities and Catholic Schools Office (Hunter New England region) supporting the implementation of the program.
	· Expert advisory group consisting of health promotion practitioners, School Education Directors, local Principals and teachers, academics with experience working with schools, parent representatives and dietitians supported program planning and implementation.
	· Regional school Directors of both Government and Catholic schools disseminated a “guiding principles” document recommending all schools in their jurisdiction to implement a vegetable and fruit break.
	· Regional and cluster school Directors advocated for the introduction of such breaks at Principal network meetings and with individual school Principals during their school visits.
	· Presentations at school Principal cluster meetings to promote the program by program staff.
	· Recruitment of “school champions”, a staff member within each school that will take responsibility for implementation.
	·Individual school specific vegetable and fruit break policy- To support, establish and sustain the program schools developed a vegetable and fruit break policy outlining how the program will be implemented and monitored and strategies for ensuring no child misses out due to financial difficulties.
Staff training & professional development	· 1-day (6 hour) ‘healthy eating’ workshop for school champions held across the Hunter New England region. Fifteen workshops were held across the region to allow maximum access by schools.
	· 1-day teacher relief funding (AU$250) for small schools (less than 300 students) for “school champion” to attend training.
	· 2 hr video-conference or self-paced online module for non-attending schools.
Provision of curriculum resources and materials, and information for parents	· Resources were provided to schools: an easy-to-follow manual and a CD containing curriculum material (the same as that available for comparison schools), policy templates, information for parents, and newsletter articles (available for download http://www.goodforkids.nsw.gov.au/Parents).
Incentives	· Following certification (that is once schools developed a school policy committing to implement the program everyday in at least 80 % of classes and to ensuring that no child misses out due to financial difficulties) schools received a free water bottle for every student and teacher.
Follow-up support	· Scripted 15-minute computer-assisted telephone interviews (CATI) with school champions up to three weeks after the workshop to: assess school readiness to implement the program; identify barriers to implementing the program; support schools to overcome the barriers; and identify schools requiring further support. · If during the scripted 15 minute CATI it was determined the school required more specific support the school was offered additional support calls from a trained health promotion support officer. Schools received a maximum of two phone calls over a three-month period. The support officer called the school contact to further discuss the barriers identified in the CATI and to offer advice or resources to meet the needs of that school. · Quarterly *Good for Kids, Good for Life* newsletter e-mailed to school champion to promote the program, celebrate successes, describe case studies in local schools and review future programs and support.
Implementation performance monitoring and feedback	· A one-off tailored ‘school report’ based on the Principal’s responses to the baseline telephone interview were provided to Principals and school champions. The report identified the vegetable and fruit break policies and programs that the school had in place and recommended specific strategies, resources or support that the study could offer to improve their vegetable and fruit break. · Regional Directors and School Education Directors were provided two six-monthly reports, which described the proportions of their schools that participated in the workshops and were “certified” for vegetable and fruit breaks. Directors were asked to disseminate these results to Principals through existing communication channels such as Principal meetings and newsletters.

#### Comparison schools: routine information-based support

Comparison schools were not offered the multi-strategy intervention described above, but were offered access to information-based support provided by a non-government organization [24]. Information regarding the program was provided to schools via a website, newsletters and events. If a school chose to register for the program, teaching resource materials were forwarded to the school, with schools able to receive e-mail and telephone information-based support if desired. If the school provided evidence of having adopted the program, they were eligible to be ‘certified’ as such and to receive additional resource materials and obtain access to ongoing e-mail and telephone support. In some areas of the state, schools could access additional support provided at the discretion of local health promotion teams.

### Data collection

To assess school characteristics, study outcomes and intervention delivery, a 20-minute computer-assisted telephone interview was conducted with school Principals or their nominated delegate (hereinafter referred to as ‘Principals’). The interviews were conducted at baseline (November 2006–April 2007) and following 11 to 15 months of intervention (October 2008– March 2009). In addition, telephone surveys with school champions (nominated representatives of intervention schools) and audits of project records were conducted at follow-up to assess delivery of the intervention components in the intervention schools. The telephone survey instruments were developed, reviewed and pre-tested by an expert advisory group and pilot tested with a sample of 10 primary school Principals to assess acceptability and comprehension. Trained research assistants conducted the telephone interviews.

### Measures

#### School characteristics

During the baseline and follow-up telephone interview, Principals were asked to report the school size (number of students). Other school information including school type (Government, non-Government Catholic or non-Government Independent) and the postcode of the locality of the school were obtained from school websites.

#### Prevalence of vegetable and fruit breaks

At baseline and follow-up Principals were asked to report whether, in the preceding year, classes at their school had implemented specific breaks in classes to allow children to eat vegetables or fruit during class time (‘yes all classes’, ‘yes some classes’, ‘no classes’, ‘don’t know’).

The accuracy of Principal-reported implementation of vegetable and fruit breaks in schools was assessed in a convenience sample of intervention schools (n = 42; 10 %). Based on observations made in these schools over a 9-week period, pre-service teachers located in schools reported in a pen-paper survey if classes at the school had specific breaks or if students had permission to eat vegetables and/or fruit during class time (‘yes all classes’, ‘yes some classes’, ‘no classes’, ‘don’t know’). The pre-service teacher surveys were completed within one month of the Principal telephone survey. Comparison of Principal and pre-service teacher report of vegetable and fruit breaks revealed perfect agreement (Kappa = 1.0).

#### Delivery of the multi-strategy intervention

The telephone interviews with Principals and school champions of intervention schools, as well as project records, were used to assess delivery of the following intervention components:

##### Leadership support

During the follow-up telephone survey Principals were asked whether their school Director had discussed the implementation of healthy eating and/or physical activity programs at their school (yes, no). In addition, project records were used to determine if the school had a registered school champion (yes, no).

##### Staff attendance at training

Project records were used to determine if a teacher of the school had attended the workshop training or participated in the 2 hr video conference (yes, no).

##### Receipt of the program resources and materials

School champions were asked during their 15-minute follow-up computer-assisted telephone interview (CATI) support call if they had received the resource folder (yes, no).

##### Receipt of scheduled follow-up telephone support calls

Project records were used to determine the number of school champions who accepted the follow-up CATI support call and additional calls from the school support officer.

##### Receipt of tailored feedback report

School champions were asked during their follow-up computer-assisted telephone interview (CATI) support call if they had received the tailored school report (yes, no).

### Analyses

All analyses were conducted with the statistical package SAS Version 9.2 (SAS Institute Inc., Cary, NC, USA). The reported number of enrolled students in each school was used to categorize school size as: ‘small’ (1–159 students); ‘medium’ (160–450 students); or ‘large’ (451+ students). Schools with postcodes ranked socio-economically in the top 50 % of New South Wales [46] were categorized as schools of ‘higher socio-economic status’ while those in the lower 50 % were categorized as schools of ‘lower socio-economic status’. School postcode areas were also used to categorize the school’s locality as either ‘rural’ (those schools in outer regional, remote and very remote areas), or ‘urban’ (those in regional cities and inner regional areas) [47]. The prevalence of vegetable and fruit breaks within schools was calculated as the proportion of all Principals reporting that ‘all or some’ classes had such a break. McNemars test was used to ascertain whether there was a statistically significant change (p-value ≤ 0.05) in the proportion of intervention and comparison schools implementing vegetable and fruit breaks at baseline and follow-up. A Generalized Estimating Equation (GEE) approach [48] controlling for baseline values, was used to examine the change in the prevalence of vegetable and fruit breaks in intervention schools compared to comparison schools. Differences were considered significant if the interaction term between experimental condition and time had a p-value ≤ 0.05. To assess the potential impact of selective non participation bias due to study attrition, an additional analysis was conducted whereby all schools lost to follow-up were included in the GEE model (using last value carried forward method). Further GEE subgroup analyses were undertaken to determine if implementation of vegetable and fruit breaks differed between intervention and comparison schools according to school type, size, rurality or socioeconomic area.

## Results

### Sample and school characteristics

Among intervention schools, 407 eligible schools completed baseline data collection (96.4 % response rate). Of these schools, 388 (95.3 %) provided follow-up data. Among comparison schools, 316 of eligible schools completed baseline data collection (77.8 % response rate), and of these, 258 (81.6 %) provided follow-up data (Figure 1). Compared to schools that only completed the baseline survey, schools providing data at both time points were significantly more likely to be government, rural and small schools (p < 0.05).

Relative to comparison schools, intervention schools were more likely to be small, and located in rural and lower socio-economic areas (Table 2). Of the participants who completed the survey, 518 (80.2 %) were Principals, 6.8 % were deputy or assistant Principals, 7.7 % were acting Principals and 5.2 % had another role in the school. Participants had been employed in their current role in the school for 4.6 years (SD 4.2 years) on average.

**Table 2:** Characteristics of schools that completed baseline and follow-up interviews

**School characteristic**	**Intervention**	**Comparison**	***p*value**
	**(N = 388)**	**(N = 258)**	
	**n (%)**	**n (%)**	
**School type**			
· Government	298 (76.8)	199 (77.1)	0.923
· Non-government	90 (23.20)	59 (22.9)	
**School size**			
· Small	180 (46.9)	85 (34.6)	0.003*
· Medium	160 (41.7)	115 (46.8)	
· Large	44 (11.5)	46 (18.7)	
**ARIA**			
· Urban	233 (60.1)	198 (76.7)	<0.001*
· Rural	155 (40.0)	60 (23.3)	
**SEIFA**			
· High	100 (25.8)	109 (42.3)	<0.001*
· Low	288 (74.2)	149 (57.8)	

* Significant where alpha = 0.05; ARIA, Accessibility/Remoteness Index of Australia; SEIFA, Socio-Economic Indexes For Australia.

### Prevalence of vegetable and fruit breaks

At baseline there was no significant difference in the prevalence of vegetable and fruit breaks between intervention and comparison schools (50.3 % vs. 45.4 % respectively, p = 0.187). At follow-up, the prevalence of such breaks in intervention schools increased by 31.7 % to 82.0 % (p < 0.001) and also increased for comparison schools by 15.5 % to 60.9 % (p < 0.001) (Table 3). There was a significant group by time interaction, such that intervention schools had 2.36 times (95 % C.I. 1.60-3.49, p < 0.001) the odds of having a vegetable and fruit break compared to comparison schools at follow-up (Table 3). The effect remained significant where baseline values were carried forward for schools that did not provide follow-up data (OR 1.91; 95 % CI 1.47 – 2.48, p <0.001). The intervention effect size (OR > 2) was similar across all subgroups (P=0.031- <0.001) (Table 3).

**Table 3:** Prevalence and odds ratios of vegetable and fruit breaks for all schools and by subgroup

	**Prevalence of vegetable and fruit breaks**^**a**^				**Odds Ratio (OR)**		**p-value**
	**Intervention**		**Comparison**		**Intervention**	**Comparison**	
	**n (%)**		**n (%)**		**(95 % CI)**		
	**Baseline**	**Follow-up**	**Baseline**	**Follow-up**			
**All Schools**	195 (50.3)	318 (82.0)	116 (45.4)	157 (60.9)	2.36 (1.60-3.49)	1	<0.001*
**School Type**							
· Government	152 (51.0)	252 (84.6)	89 (45.0)	125 (62.8)	2.52 (1.60-3.97)	1	<0.001*
· Non-Government	43 (47.8)	66 (73.3)	27 (45.8)	32 (54.2)	2.14 (0.98-4.68)	1	0.057
**School size**							
· Small	76 (42.2)	138 (76.7)	34 (40.5)	51 (60.0)	2.0 (1.08-3.72)	1	0.029*
· Medium	94 (58.8)	140 (87.5)	57 (45.6)	74 (64.4)	2.68 (1.44-4.97)	1	0.002*
· Large	23 (52.3)	36 (81.8)	18 (39.1)	26 (56.5)	2.03 (0.69-6.00)	1	0.200
**ARIA**							
· Rural	55 (36.0)	118 (77.1)	29 (50.9)	42 (72.4)	2.55 (1.24-5.26)	1	0.011*
· Urban	140 (59.6)	200 (85.1)	87 (43.5)	115 (57.5)	2.13 (1.30-3.48)	1	0.003*
**SEIFA**							
· Low	136 (47.2)	234 (81.3)	70 (47.0)	97 (65.1)	2.30 (1.43- 3.71)	1	<0.001*
· High	59 (59.0)	84 (84.0)	46 (42.2)	60 (55.1)	2.18 (1.08-4.40)	1	0.031*

^*a*^A time in class for children to consume a piece of vegetable or fruit they had brought from home; * Significant where alpha = 0.05; ARIA, Accessibility/Remoteness Index of Australia; CI, Confidence Interval; SEIFA, Socio-Economic Indexes For Australia.

#### Delivery of the intervention

Table 4 shows the proportion of intervention schools that received each of the implementation strategies. Most schools (96.6 %) had registered a school champion and 95.9 % of school champions completed the follow-up CATI support call, and 100 % received an additional school support officer telephone call. The receipt of the other intervention strategies varied from 69.6 % to 30.9 %. No staff attended the 2-hour video conference.

**Table 4:** Extent of delivery of multi-strategy intervention to intervention schools

**Schools receiving intervention strategies**	**n**	**%**
Consensus process, leadership support and endorsement		
· Discussed healthy eating with school Director ^a^	250/360	69.4
· School champions registered	375/388	96.6
Staff training		
· School teacher attended training workshop	270/388	69.6
· Attended 2 hour video conference	0/388	0
Resources		
· Received resource folder ^b^	247/372	66.4
Follow-up support		
· Completed follow-up CATI call	372/388	95.9
· Received additional school support officer call ^c^	68/68	100
Program performance		
· Received tailored school reports ^b^	115/372	30.9

^*a*^*sample size varies as question not relevant for Independent schools (n = 360);*^b^*sample size varies as only those schools who completed the follow-up CATI were asked this question (n = 372);*^*c*^*sample size varies as this strategy was only offered to schools who were identified as needing additional telephone support*.

## Discussion

To our knowledge, this is the first controlled evaluation of an intervention to facilitate the implementation of a vegetable and fruit program across a large number of schools. The findings suggest that the multi-strategy intervention involving leadership support, staff training and telephone follow-up support, was effective in increasing the uptake of vegetable and fruit breaks in schools compared to more minimal, information-based support. These findings provide support for the use of a multi-strategic intervention approach in facilitating a large number of schools to implement vegetable and fruit programs across a large and diverse geographic region.

The effect size reported in the trial (approximately 15 % relative to comparison schools) was consistent with those reported in controlled trials of interventions designed to assist large numbers of schools implement health promotion programs generally (13 %-45 %) [31,34,35,49,50]. On a population wide basis, a 15 % increase in the number of schools implementing vegetable and fruit programs is likely to represent a meaningful contribution to improving public health nutrition. For example, in this trial, a 15 % increase resulted in an additional 58 schools implementing a vegetable and fruit break in most or all classes. Assuming an average school size of 250 students, almost 15,000 additional children may have benefitted from the program.

Despite the significant finding, a number of opportunities exist to further enhance the intervention. First, although 70 % of schools received training, in most schools only a single staff member was trained. Due to poor promotion and limited school access to facilities videoconference training was not attended by staff from any school. As the knowledge, skills and support of all staff are important predictors of program implementation [29,51] additional strategies targeting training of multiple staff, for example as part of staff orientation or routine professional development workshops [36,52,53], have the potential to increase program implementation. Second, while 95 % of schools received the initial follow-up CATI support call, more intensive and prolonged follow-up support has been suggested to yield improved rates of program adoption [29,53]. Third, less than 30 % of schools reported receiving the tailored school implementation feedback report. Anecdotal evidence indicated that mailed resources were frequently misplaced or unopened at schools. Further development and analysis of the most effective format and delivery of performance feedback reports is required to maximize the previously reported positive impact of such a strategy on program implementation [29,31].

A number of the study characteristics should be considered when interpreting the study findings. Given the challenges of random assignment in large complex public health interventions [54], the trial utilized a quasi-experimental design. Nonetheless, the internal validity of the trial would have been strengthened had a random experimental evaluation design been employed. The intervention was undertaken as part of a larger multi-setting child obesity prevention program [40]. The existence of the broader program may have enhanced the salience of obesity prevention efforts in the intervention community and pre-disposed schools to the implementation of obesity prevention programs such as vegetable and fruit breaks. The trial was also reliant on the self-reported existence of vegetable and fruit breaks collected as part of a telephone survey. Whilst attempts were made to validate the measure. The use of alternative survey modalities, such as the web may reduce the risk of social desirable responding [55], and the use of direct observation would represent a more objective assessment of program adoption. Additionally, schools lost to follow-up were significantly different from those that completed the survey with respect to school type, size and geographic location. This may have introduced bias due to selective non-participation. The intervention effect size, however, was similar across subgroups suggesting that risk of such bias is likely to be minimal. Finally, the intervention encouraged schools to identify strategies to ensure that all children participated in the vegetable and fruit break. While data regarding student participation was not collected in the trial, anecdotally, Principal’s frequently reported school wide participation by all students through school provision of vegetable or fruits to the small number of children unable to bring these foods from home, or by cutting up the vegetables and fruit provided by students and sharing it with all class members.

## Conclusion

The study provides novel information for public health policy makers and practitioners regarding strategies to facilitate the implementation of health promotion programs broadly, and vegetable and fruit break programs specifically. Given that maintenance of the intervention effect is expected to decrease over time [29,56], longer-term follow-up of these schools appears warranted in order to determine whether the intervention impact is sustained. Furthermore, examination of the cost effectiveness of such strategies in achieving program implementation may assist policy makers and practitioners to most effectively allocate scarce health resources to improve the health of the community.

## Competing interests

The authors declare that they have no competing interests.

## Authors’ contributions

NN, CB, PJM, and JW designed the intervention; NN, CB and RW conducted the research; LW, CB, PJM, RS, JW, AJM and DH provided overall advice on the evaluation design of the G*ood for Kids. Good for Life* program; NN, LW, CB, RW, PJM, RS, MB contributed to acquisition of data; NN, LW, CB, MB, JW analyzed and interpreted data; NN led the development of the manuscript and all authors contributed to drafts and read and approved the final manuscript.

## Pre-publication history

The pre-publication history for this paper can be accessed here:

http://www.biomedcentral.com/1471-2458/12/651/prepub
